# Alternative Splicing of Circadian Clock Genes Correlates With Temperature in Field-Grown Sugarcane

**DOI:** 10.3389/fpls.2019.01614

**Published:** 2019-12-23

**Authors:** Luíza L. B. Dantas, Cristiane P. G. Calixto, Maira M. Dourado, Monalisa S. Carneiro, John W. S. Brown, Carlos T. Hotta

**Affiliations:** ^1^ Departamento de Bioquímica, Instituto de Química, Universidade de São Paulo, São Paulo, Brazil; ^2^ Division of Plant Sciences, School of Life Sciences, University of Dundee at the James Hutton Institute, Dundee, United Kingdom; ^3^ Departmento de Biotecnologia, Produção Vegetal e Animal, Centro de Ciências Agrícolas, Universidade Federal de São Carlos, Araras, Brazil; ^4^ Cell and Molecular Sciences, The James Hutton Institute, Dundee, United Kingdom

**Keywords:** alternative splicing, circadian clock, diel rhythms, field experiment, gene expression, sugarcane

## Abstract

Alternative Splicing (AS) is a mechanism that generates different mature transcripts from precursor mRNAs (pre-mRNAs) of the same gene. In plants, a wide range of physiological and metabolic events are related to AS, as well as fast responses to changes in temperature. AS is present in around 60% of intron-containing genes in *Arabidopsis*, 46% in rice, and 38% in maize and it is widespread among the circadian clock genes. Little is known about how AS influences the circadian clock of C4 plants, like commercial sugarcane, a C4 crop with a complex hybrid genome. This work aims to test if the daily dynamics of AS forms of circadian clock genes are regulated by environmental factors, such as temperature, in the field. A systematic search for AS in five sugarcane clock genes, *ScLHY*, *ScPRR37*, *ScPRR73, ScPRR95,* and *ScTOC1* using different organs of sugarcane sampled during winter, with 4 months old plants, and during summer, with 9 months old plants, revealed temperature- and organ-dependent expression of at least one alternatively spliced isoform in all genes. Expression of AS isoforms varied according to the season. Our results suggest that AS events in circadian clock genes are correlated with temperature.

## Introduction

During gene expression in a eukaryotic cell, pre-mRNAs undergo splicing to remove introns and join exons in a mature transcript, generating an open reading frame (ORF) for protein synthesis. Splicing is largely co-transcriptional in yeast, *Drosophila melanogaster*, mammals and *Arabidopsis thaliana* (L.) Heynth. (Beyer and Osheim, 1988; Oesterreich et al., 2016; Saldi et al., 2016; Godoy Herz et al., 2019; Jabre et al., 2019). Alternative splicing (AS) is a mechanism that generates different RNAm transcripts from a single gene. As a result of AS, the mature mRNAs represent another level of gene expression regulation at the post-transcriptional level by, for example, insertion of premature termination codons (PTC), which can target some AS isoforms to degradation by the nonsense-mediated mRNA decay (NMD) pathway (Filichkin and Mockler, 2012; Kalyna et al., 2012; Marquez et al., 2012). Alternatively, those transcripts carrying PTCs could produce truncated polypeptides missing functional domains and motifs that can compete with the corresponding functional protein (Seo et al., 2011; Mastrangelo et al., 2012; Reddy et al., 2013). In addition, AS can increase protein by producing mRNAs from the same gene that encode protein variants with different diversity in function, localization, and stability (Syed et al., 2012; Chaudhary et al., 2019). AS is a ubiquitous process observed from *Drosophila* to humans and plants (Graveley, 2005; Filichkin et al., 2010; Marquez et al., 2012; Kornblihtt et al., 2013). In plants, a wide range of physiological and metabolic events and responses are related to AS. This mechanism is so widespread that it was reported in more than 60% of intron-containing genes in *Arabidopsis*, 46% in rice, and 38% in maize (Zhang et al., 2010; Marquez et al., 2012; Staiger and Brown, 2013a; Thatcher et al., 2014; Chamala et al., 2015; Filichkin et al., 2015b; Min et al., 2015). There is evidence of organ and tissue-specific alternative transcript forms and even alternative transcript isoforms in different subcellular locations (Nagashima et al., 2011; Kriechbaumer et al., 2012; Remy et al., 2013; Vaneechoutte et al., 2017). AS impacts development, from the early gametic cell specification to the seed maturation (Moll et al., 2008; Liu et al., 2009; Sugliani et al., 2010; Fouquet et al., 2011; Zhang et al., 2016; Szakonyi and Duque, 2018) and even flowering time and floral development (Zhang et al., 2011; Severing et al., 2012; Rosloski et al., 2013). Both biotic and abiotic stress responses are also closely related to AS (Staiger and Brown, 2013a; Shang et al., 2017; Wang et al., 2018). Plants under stress conditions change their AS patterns dramatically (Palusa et al., 2007; Staiger and Brown, 2013a; Ding et al., 2014; Filichkin et al., 2015a; Calixto et al., 2018; Calixto et al., 2019). Also, many circadian clock genes generate alternative transcript forms with PTCs under different environmental conditions (Filichkin et al., 2010; James et al., 2012c; James et al., 2012a; Jones et al., 2012; Filichkin et al., 2015a; Calixto et al., 2016). The presence of alternative transcripts in the circadian clock genes is highly conserved among different plant species, such as *Arabidopsis*, *Populus alba* L., *Brachypodium distachyon* (L.) P. Beauv., and rice (*Oryza sativa* L.)—all C3 plants (Filichkin and Mockler, 2012). Little is known about how AS influences the circadian clock of C4 plants.

The circadian clock is a 24 h endogenous timekeeper mechanism that anticipates the Earth’s day/night and seasonal cycles (Hsu and Harmer, 2014; Millar, 2016; McClung, 2019). Like AS, the circadian clock is associated with growth, photosynthesis, and biomass in plants, so these two regulatory mechanisms may act together, or even regulate each other (Dodd et al., 2005; Lu et al., 2005; Harmer, 2009; Lai et al., 2012; Syed et al., 2012; Staiger and Brown, 2013a). The circadian clock consists of multiple interlocked transcription–translation feedback loops connected with input pathways that feed the circadian clock function with environmental cues, such as light and temperature, and with output pathways that are responsible for coordinating several major metabolic and physiological processes (Pokhilko et al., 2012; Haydon et al., 2013; Hsu and Harmer, 2014). In *Arabidopsis*, the main loop consists in three different components: *CIRCADIAN CLOCK ASSOCIATED* 1 (*CCA1*), *LATE ELONGATED HYPOCOTYL* (*LHY*), expressed around dawn and *TIMING OF CHLOROPHYLL* A/B *BINDING PROTEIN 1* (*TOC1*), expressed around dusk (Alabadí et al., 2001). Closely associated with this loop are the PSEUDO-RESPONSE *REGULATORS 7*, *3* and *9* (*PRR7*, *PRR3*, *PRR9*) (Locke et al., 2005; Zeilinger et al., 2006; Para et al., 2007; Nakamichi et al., 2010). The components of the central loop and the associated PRRs are conserved among other plant species, including crops like rice, maize (*Zea mays* L.), barley (*Hordeum vulgare* L.) and sugarcane (*Saccharum* hybrid) (Murakami et al., 2007; Khan et al., 2010; Hotta et al., 2013; Calixto et al., 2015). The sugarcane circadian clock, although sharing conserved components with other plants, may have a broader influence over sugarcane physiology, with 32% of sugarcane transcripts showing rhythms under circadian conditions (Hotta et al., 2013).

Sugarcane is a C4 grass that stores large amounts of sucrose in its stems, which can reach as much as 700 mM or 50% of the culm dry weight (Moore, 1995). Its genome is exceptionally complex, showing aneuploidy and a massive autopolyploidy that can range from six to fourteen copies of each chromosome (Garcia et al., 2013). The genome size of commercial modern sugarcane is estimated to be around 10 Gb (de Setta et al., 2014; Chan et al., 2018). Because modern sugarcane cultivars are interspecific hybrids progenies from *Saccharum officinarum* L. and *Saccharum spontaneum* L., about 80% of sugarcane chromosomes comes from *S. officinarum*, 10% comes from *S. spontaneum* and 10% are recombinants of these two species (D’Hont et al., 1996; Cuadrado et al., 2004; D’Hont, 2005). Sugarcane is a valuable commodity, responsible for 80% of sugar and 40% of ethanol worldwide (FAO, 2015). The remaining biomass from sugarcane can also be used for bioenergy production: the bagasse can be either burned to generate electricity or have its cell wall hydrolyzed to yield simple sugars, which can be fermented to produce second-generation biofuel (Amorim et al., 2011).

Although a great deal of data has been generated about the plant circadian clock, sugarcane, and AS, the majority of these studies have been performed under highly controlled experimental conditions. Such conditions are essential for reproducibility and, for the circadian clock, a constant environment is one way to demonstrate the inner mechanism generating self-sustained rhythms, as well as rhythmic responses. However, those conditions are far from the environment that crops face in nature, with fluctuations and complex interactions between abiotic and biotic variables (Annunziata et al., 2017; Annunziata et al., 2018; Shalit-Kaneh et al., 2018). In order to better understand the relationship between the circadian clock and AS and how this relationship impacts on crops, it is essential to expand experiments to field conditions. Indeed, essential in-field studies using *Arabidopsis* (Richards et al., 2012; Annunziata et al., 2018) and rice (Izawa et al., 2011a; Sato et al., 2011; Nagano et al., 2012) show that the complex natural cyclic environment has a broader impact on rhythmic gene expression. So far, no studies have approached the AS profile on circadian clock genes under such conditions.

In this study, we examined whether the daily dynamics of AS forms of circadian clock genes are regulated by environmental factors in the field. We used sugarcane organs extracted from field-grown plants when individuals were 4-months-old, during the Brazilian winter, and 9-months-old, during the Brazilian summer. We investigated the AS profile of sugarcane circadian clock genes in this fluctuating natural environment. Data shows that there is at least one alternatively spliced form for each of the five circadian clock genes analyzed. During winter, when temperatures are lower, alternative transcripts are more highly expressed than in summer, with higher temperatures, which suggests that AS might be related to the fluctuating environmental temperature in the field. The different organs also showed different levels of AS and leaf has most of the diversity in AS events. Collectively, our data suggest temperature correlates with AS in the circadian clock of sugarcane plants grown in a natural environment, possibly as a mechanism of dynamic adjustment of the circadian clock.

## Material and Methods

### Field Conditions and Plant Harvesting

The sugarcane field where the experiment was conducted was located at the Federal University of São Carlos, campus Araras, in São Paulo state, Brazil (22°21′25″ S, 47°23′3″ W, at an altitude of 611 m). The soil of the site was classified as a Typic Eutroferric Red Latosol. Sugarcane tillers from the commercial variety SP80-3280 (*Saccharum* hybrid) were planted in soil in April/2012. Field design had 8 plots (**Figure S1A**). Each plot had 4 rows containing 20 tillers each. Only sugarcane plants from both central lines were used in order to avoid border effects. Sugarcane individuals were randomly picked from two plots in order to avoid variability of both the local environment and individual plants. Data on environmental conditions was acquired from a local weather station (**Figures S1B**, **C**). Leaves +1 (L1), a source organ and the first fully photosynthetically active leaf in sugarcane, were sampled from the selected individual plants during two different seasons, and therefore different developmental stages. In the first harvest, 4-months-old plants were sampled in August/2012, during winter; in the second harvest, 9-months-old plants were sampled in January/2013, during summer. In winter, dawn was at 6:30, and dusk was at 18:00 (11.5 h day/12.5 h night). In summer, dawn was at 5:45, and dusk was at 19:00 (13.25 h day/10.75 h night). To compare the rhythms of samples harvested in different seasons, the time of harvesting were normalized to a photoperiod of 12 h day/12 h night using the following equations: for times during the day: ZT = 12*T*P_d_^‑1^, where ZT is the normalized time, T is the time from dawn (in hours), and P_d_ is the length of the day (in hours); for times during the night: ZT = 12 + 12*(T ‑ P_d_)*P_n_^‑1^, where ZT is the normalized time, T is the time from dawn (in hours), P_d_ is the length of the day (in hours), and P_n_ is the length of the night (in hours). Because the 9-month-old plants had their culms fully developed, internodes 1 and 2 (I1) and internode 5 (I5) were also sampled. Both internodes are sink tissues with different profiles: internodes 1 and 2 mostly undergo intense cell division and elongation, whereas internode 5 undergoes sucrose storage. For every time point, 9 individuals were randomly selected in the assigned plots and harvested from the culm up. After that, those 9 individuals were separated into three pools of three individuals, each pool formed a biological replicate and then their leaves +1 were extracted. For all harvests, plants were sampled every 2 h for 26 h, starting 2 h before dawn. In total, the time course consisted of 14 time points in each harvest/season. After every time point sampling, a process that took less than 30 min on average, tissue was immediately frozen in liquid nitrogen.

### RNA Extraction

Sugarcane leaves previously frozen in liquid nitrogen were pulverized using dry ice and a grinder. Then, 100 mg of this ground tissue was used for total RNA extractions using Trizol (Life Technologies, Carlsbad, CA, USA), followed by treatment with DNase I (Life Technologies, Carlsbad, CA, USA) and cleaned with RNeasy Plant Mini Kit (QIAGEN, Valencia, CA, USA). The quality and quantity of each RNA sample were checked using an Agilent RNA 6000 Nano Kit Bioanalyzer (Agilent Technologies, Palo Alto, CA, USA). All RNA samples were stored at ‑80°C.

### cDNA Synthesis

cDNA was synthesized using SuperScript III First-Strand Synthesis System for RT-PCR (Life Technologies, Carlsbad, CA, USA) starting from 5 μg of total RNA. For all reactions, both Oligo(dT) and Random Hexamers primers were used. All cDNA samples were stored at ‑20°C.

### PCR Reactions

Primers used in PCR reactions were designed using the software PrimerQuest Tool (IDT) (http://www.idtdna.com/primerquest/home/index). Each pair of primers was gene-specific and amplified fragments ranging from 242 bp to 805 bp (**Table S1**). All PCR reactions were carried out using Go Taq DNA Polymerase (Promega, Madison, WI, USA) and following the manufacturer’s protocol. Briefly, each 20-μl PCR reaction contained 2 μl of template, 10 μM of each primer, 4 μl of 5x Green Go Taq Buffer, 0.15 μl of Go Taq DNA Polymerase, 2 mM of dNTPs. PCR conditions were: an initial step at 94°C for 2 min, followed by 20 – 30 cycles of 94°C for 15 s, 50°C for 15 s, 72°C for 30 s, followed by a final extension of 72°C for 5 min. PCR reactions using primers amplifying control genes *ScGAPDH* and *ScPP2AA2* were performed for all cDNA samples. Reactions containing negative control using RNA as template and positive control using genomic DNA as template were carried out. All PCR-amplified fragments were analyzed by taking 10 μl of reaction and run on an electrophoresis gel of 1.5% agarose (Life Technologies, Carlsbad, CA, USA) and 1x TBE (50 mM Tris–HCl pH 8, 50 mM Boric Acid, 1mM EDTA).

### High-Resolution RT-PCR

High-Resolution RT-PCR (HR RT-PCR) reactions were performed based on Simpson et al. (2007) and Simpson et al. (2019). For all reactions, the forward primer was labeled with 6-carboxyfluorescein (FAM). Reactions consisted of a final volume of 20 μl which had 2 μl of cDNA, 10 μM of each primer, 2 μl of 10x PCR Reaction Buffer with MgCl2 (Roche Life Science, Indianapolis, IN, USA), 0.15 μl Taq DNA Polymerase (Roche Life Science, Indianapolis, IN, USA), and 2 mM of dNTPs. The PCR detailed program was: an initial step at 94°C for 2 min, followed by 22–26 cycles of 94°C for 15 s, 50°C for 15 s, 70°C for 30 s, followed by a final extension of 70°C for 5 min. Once PCR reactions were complete, 1 μl of each reaction was added to a mix containing 9 μl of Hi-Di Formamide (Applied Biosystems, Life Technologies, Carlsbad, CA, USA) and 0.5 μl of GeneScan 500 LIZ Size Standard (Applied Biosystems, Carlsbad, CA, USA). The RT-PCR products were separated on an ABI 3730 Automatic DNA Sequencer (Applied Biosystems, Life Technologies, Carlsbad, CA, USA). The results were analyzed using GeneMapper fragment analysis software (Applied Biosystems, Carlsbad, CA, USA). LOESS (locally estimated scatterplot smoothing) regression was used to detect trends in the data. The maximum value of the LOESS curve between ZT0 and ZT22 was considered the peak of the rhythm. The code to fully reproduce our analysis is available on GitHub (https://github.com/LabHotta/AlternativeSplicing) and archived on Zenodo (http://doi.org/10.5281/zenodo.3509232).

### Cloning and Sequencing

In order to identify alternatively spliced forms, as well as differentially expressed alleles, RT-PCR fragments were cloned and sequenced. For this, PCR fragments were purified using the QIAquick PCR Purification Kit (QIAGEN, Valencia, CA, USA). Each purified fragment was cloned into pGEM-T Easy Vector (Promega, Madison, WI, USA) following the manufacturer’s protocol. Briefly, each reaction contained 3 μl of purified PCR product, 5 μl of Rapid Ligation Buffer, T4 DNA Ligase, 1 μl of pGEM-T Easy Vector (50 ng) and 1 μl T4 DNA Ligase (3 Weiss units/μl). Ligation reactions were incubated overnight at 4°C. Two micro-liter of each ligation reactions were used for heat-shock transformation of 50 μl of JM109 High-Efficiency Competent Cells (Promega, Madison, WI, USA), following manufacturer’s instructions. Transformed cells were plated on LB/ampicillin/IPTG/X-gal media and incubated overnight at 37°C. Random colonies were selected to use in plasmid extraction using QIAprep Miniprep Kit (QIAGEN, Valencia, CA, USA). Positive plasmids were confirmed by PCR reactions, digestions using restriction enzymes *PstI* and *Nco1* (Promega, Madison, WI, USA) and Sanger sequencing. In order to identify alternative splicing events and single-nucleotide polymorphisms, results were compared to sugarcane genomic sequences, and sugarcane transcripts from Sugarcane Assembled Sequences (SAS) from SUCEST (http://sucest-fun.org/) using Clustal Omega (https://www.ebi.ac.uk/Tools/msa/clustalo/). *In silico* translation was carried out using ExPaSy translation tool (http://web.expasy.org/translate/).

## Results

### Identification of Alternatively Spliced Forms of Sugarcane Circadian Clock Genes

**Figure 1:**
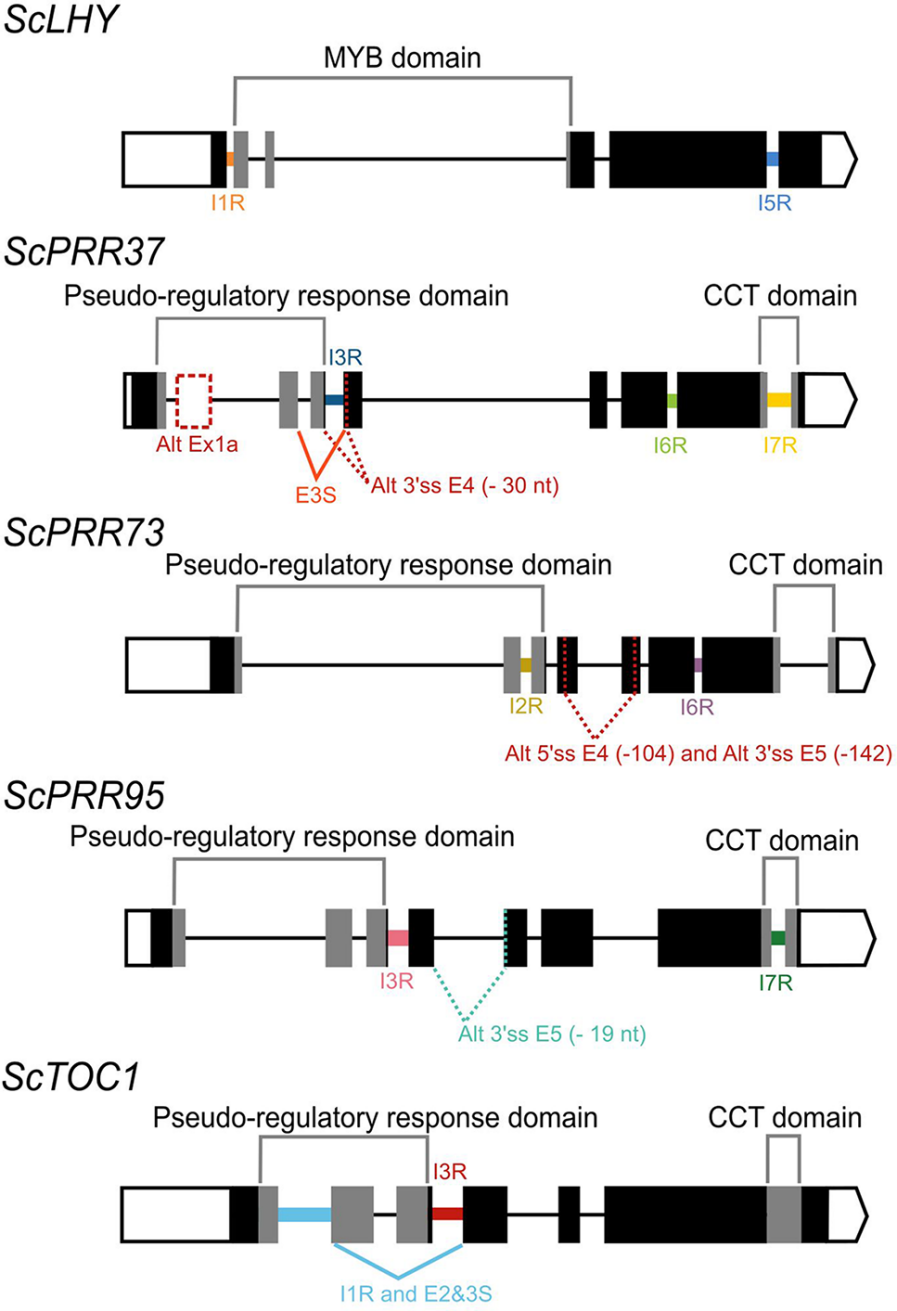
Alternative splicing events identified in the sugarcane circadian clock genes. Alternative splicing (AS) events are shown in the gene structure of *ScLHY*, *ScPRR37*, *ScPRR73*, *ScPRR95,* and *ScTOC1*. White boxes show 5′ and 3′ UTR regions; black boxes show exons; black lines represent introns. The main protein domains are in grey. AS events are shown in colored solid lines, colored dotted lines or in dotted white boxes and the splicing events are illustrated below each event. Different colors were chosen to mark the AS events investigated later, whereas events that were not investigated further are in dark red. IR, intron retention; ES, exon skipping; Alt 5′ ss, alternative 5′ splice sites; Alt 3′ ss, alternative 3′ splice sites; Alt Ex, alternative exon.

In *Arabidopsis*, alternative splicing (AS) events of the circadian clock genes are well known (James et al., 2012b). In order to investigate AS of sugarcane circadian clock genes, it is important to first determine and annotate their genomic sequences/structures. We first described the gene structure of previously identified homologs *ScLHY*, *ScPRR73*, *ScPRR95* and *ScTOC1* (Hotta et al., 2013). These homologs were identified by Hotta et al. (2013) using BLAST searches of *Arabidopsis* and rice circadian clock coding DNA sequence (CDS), on the sugarcane Expressed Sequence Tags (ESTs) collection database at SUCEST (http://sucest-fun.org/). This study identified only CDS sequences of sugarcane circadian clock genes but not their genomic sequences due to the relatively few genomic sequences publicly available at that time for the sugarcane cultivar of interest, plus a high percentage of incomplete genomic sequences (Vicentini et al., 2012; de Setta et al., 2014). Here we used the SUCEST sequences (Hotta et al., 2013) to search for their genomic sequences in an unpublished sugarcane genomic database (Souza et al., 2019) We identified genomic contigs for *ScLHY* (previously identified as *ScCCA1*), *ScTOC1*, *ScPRR73* (previously identified as *ScPRR3*) and *ScPRR95* (previously identified as *ScPRR59*) (**Table S2**). A new homolog, *ScPRR37*, was identified here for the first time in sugarcane (**Table S2**, **Figure 1**). The sugarcane genes were annotated by comparison with genomic sequences of circadian clock homologs from barley and sorghum (Calixto et al., 2015). The exon/intron structures are shown in **Figure 1**; introns contained the canonical GT–AG dinucleotides at the exon-intron boundaries. The putative CDS of each gene analyzed was translated *in silico* in order to confirm intact open reading frames (ORFs).

To identify AS transcripts, RT-PCR was performed on cDNA synthesized from L1 RNA, collected at three different time points, which corresponded to the highest gene expression for each clock gene, based on the findings from Hotta et al. (2013). RT-PCR used pairs of primers to generate overlapping amplicons to cover the full length of the transcripts for each sugarcane circadian clock genes, *ScLHY*, *ScPRR37*, *ScPRR73*, *ScPRR95,* and *ScTOC1*. RT-PCR products were cloned and sequenced, which confirmed the annotation and identified AS events. Confirmation of different transcript isoforms was carried out by using two different approaches combined: RT-PCR product size and fragment cloning followed by sequencing.

We found evidence of AS events in all five genes analyzed (**Table 1**). Intron retention (IR) was the most frequent AS event identified, resulting in the inclusion of premature termination codons (PTC) in all cases (**Figure 1**). All five genes had at least two IR events, and *ScTOC1* had retention of the first intron (I1R) combined with the skipping of exons 2 and 3 (E23S). *ScLHY* had retention of introns 1 (I1R) and 5 (I5R). *ScPRR37* had retention of introns 3 (I3R), 6 (I6R) and 7 (I7R). *ScPRR73* had retention of introns 2 (I2R) and 6 (I6R). *ScPRR95* had two introns retained: intron 3 (I3R) and intron 7 (I7R) (**Figure 1**). All the other introns in the genes were efficiently spliced. Exon skipping (ES) was detected in *ScPRR37* (E3S) and *ScTOC1*, the latter having two exons skipped at once (E23S), combined with I1R (**Figure 1**). Alternative 3′ splice sites (Alt 3′ ss) were found in both *ScPRR37* and *ScPRR95* in exon 4 (E4) and exon 5 (E5), respectively (**Figure 1**). In *ScPRR73*, we found an AS event between exons 4 and 5 involving an alternative 5′ splice site (Alt 5′ss) in exon 4 (E4) being spliced to an Alt3′ss in exon 5. This AS event removes 104 nt and 142 nt from exons 4 and 5, respectively (total of 246 nt), but remains in frame and potentially produces a protein missing 82 amino acids compared to the wild-type protein.

**Table 1:** AS events found in the sugarcane circadian clock genes analyzed.

Gene	AS event^*^	Effect on transcript
*ScLHY*	Intron 1 retained (I1R)	Inserts a PTC on the transcript
	Intron 5 retained (I5R)	Creates a PTC on Exon 6
*ScPRR37*	Alternative exon 2 (Ex2a)	Inserts PTCs in the transcript
	Exon 3 skipped (E3S)	Inserts PTCs on Exon 4
	Intron 3 retained (I3R)	Creates PTC on Exon 3
	Intron 6 retained (I6R)	Inserts a PTC on the transcript
	Intron 7 retained (I7R)	Creates PTCs on Exon 8
	Alternative 3′ splice site in exon 4	Protein stays in frame, removes 30 nucleotides from the PRR domain
*ScPRR73*	Intron 2 retained (I2R)	Inserts a PTC on the transcript
	Alternative 5′ splice site in exon 4 and alternative 3′ splice site in exon 5	Protein stays in frame, removes 245 nucleotides from transcript
	Intron 6 retained (I6R)	Inserts a PTC on the transcript
*ScPRR95*	Alternative 3′ splice site in exon 5	Inserts a PTC on Exon 5
	Intron 7 retained (I7R)	Inserts a PTC on the transcript
*ScTOC1*	Intron 1 retained (I1R) combined with Exons 2 and 3 skipped (E23S)	Inserts a PTC on the transcript
	Exon 3 skipped (E3S)	Inserts a PTC on the transcript

^*^All AS events were confirmed by Sanger sequencing.

It is possible that some of the alternative sequences we found are not AS events but different haplotypes since sugarcane is highly polyploid and aneuploid. To exclude this possibility, we obtained the sequences for circadian clock genes from four different sugarcane sequencing projects (Riaño-Pachón and Mattiello, 2017; Garsmeur et al., 2018; Zhang et al., 2018; Souza et al., 2019). We found 11 sequences for the 5′ portion of the *ScLHY*: 4 from *S. spontaneum* (Zhang et al., 2018), 6 from the commercial *Saccharum* hybrid SP80-3280 (Riaño-Pachón and Mattiello, 2017; Souza et al., 2019) and 1 from the commercial *Saccharum* hybrid R570 (Garsmeur et al., 2018). All *ScLHY* sequences had the complete intron 1 (**Figure S2**). Similarly, all 16 sequences that had the end portion of *ScLHY* contained the complete intron 5 (**Figure S2**). All other detected AS events had 7 to 15 sequences supporting the conclusion that these alternative sequences are not the result of different sugarcane haplotypes (**Figures S3**–**S6**).

### Expressed AS Forms in Different Seasons in Sugarcane Leaves

We used HR RT-PCR (Simpson et al., 2019) to examine the daily dynamics of the expressed isoforms of the sugarcane circadian clock genes in two different seasons, winter and summer, using field-grown plants that were 4 and 9 months old, respectively. Briefly, the HR RT-PCR system uses fluorescently labelled primers to amplify across an AS event, followed by fragment analysis in an automatic DNA sequencer that quantifies the relative levels of RT-PCR products and thereby splicing ratios that reflect different splice site choices. The primers used for the HR RT-PCR assays had the same sequence of those used to amplify each gene region on RT-PCR experiments (**Table S1**). Leaf +1 (L1), internode 1 and 2 (I1), and internode5 (I5) samples, harvested every 2 h during 26 h, starting from 2 h before dawn were used. As reference genes to normalize data, we used *ScGAPDH* and *ScPP2AA2* (Iskandar et al., 2004; James et al., 2012a). As the experiments were done in different seasons, we have normalized the time of sampling to fit in a 12 h day/12 h night photoperiod such that ZT00 is set to dawn, and ZT12 is set to dusk.

**Figure 2:**
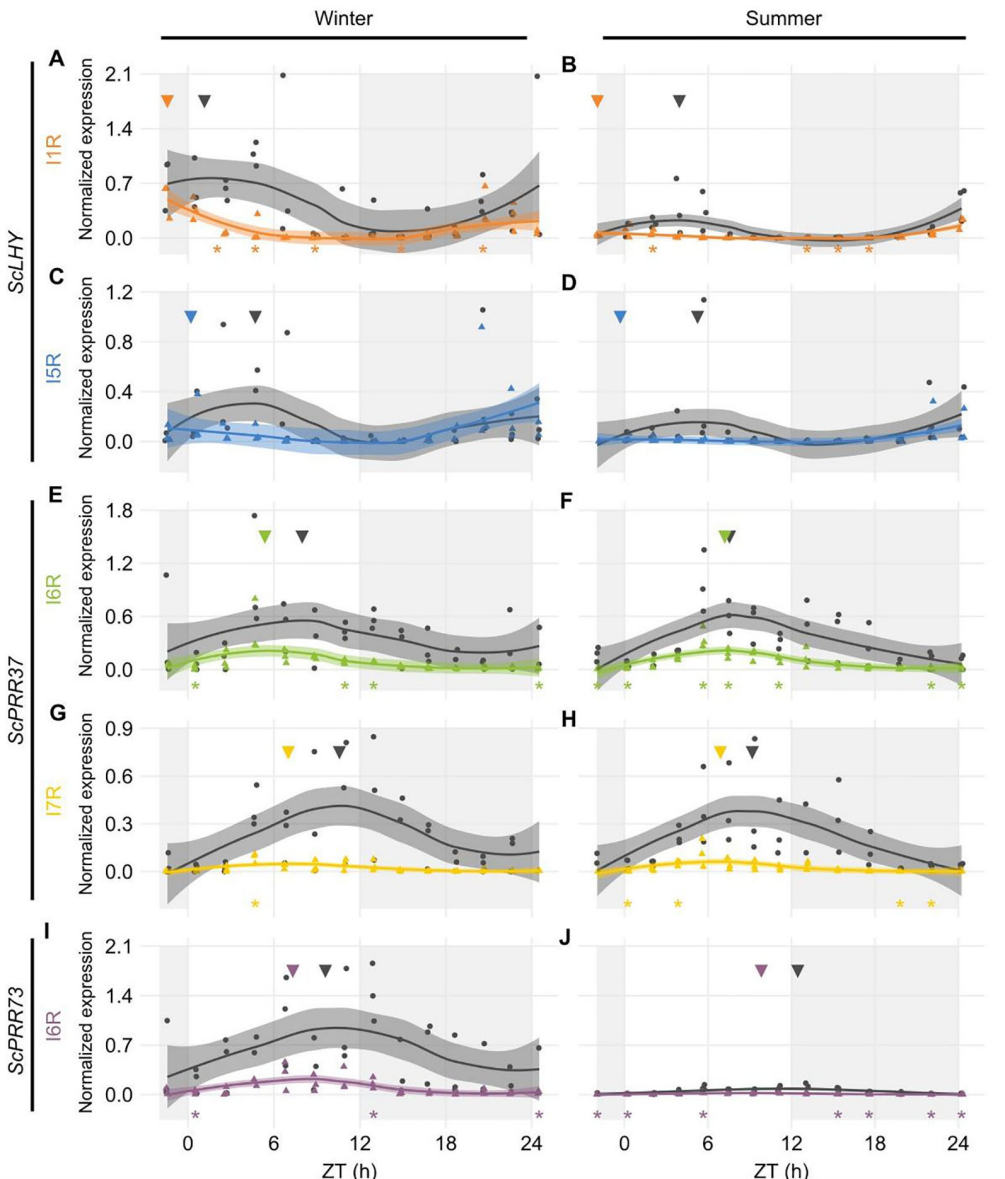
Diel expression profile of fully spliced and alternative transcript isoforms in different seasons. Biological replicates (circles and triangles) and their LOESS curve (continuous lines ± SE) of fully spliced (FS, black) and alternative transcript forms (AS, colored) for the winter samples (4-month-old plants, left) the summer samples (9-month-old plants, right). **(A**, **B)** *ScLHY* gene expression shows levels of I1R (orange) and **(C**, **D)** I5R events (blue). **(E, F)** *ScPRR37* gene expression shows levels of I6R (green), and **(G**, **H)** I6R (yellow); **(I**, **J)** *ScPRR73* gene expression shows levels of I2R (purple). Inverted triangles show the time of the maximum value of the LOESS curve. The light-gray boxes represent the night period. Statistical significance was analyzed by paired Student’s t-test, ^*^p < 0.05.

From the five sugarcane circadian clock genes analyzed, HR RT-PCR experiments detected high levels of AS in *ScLHY*, *ScPRR37,* and *ScPRR73* (**Figure 2**), but not in *ScPRR95* and *ScTOC1*, in L1 (**Figure S7**). In general, the AS isoform peaked earlier than the FS form in 8 of the 9 conditions assayed (**Figures 2A**–**I**), apart from *ScLHY,* where the FS forms peaked at the same normalized time in winter and summer samples.

*ScLHY* had confirmed events of intron 1 and 5 retention (I1R and I5R) in both harvests (**Figures 2A**–**D**). The peak of expression of the *ScLHY* alternative isoforms did not match the peak of expression of the fully spliced functional (FS) isoform. *ScLHY* I1R peaked close to dawn in winter and summer samples, while the FS for this region peaked one hour after dawn in winter plants (ZT01) and five hours after dawn (ZT05) in summer plants (**Figures 2A**, **B**). *ScLHY* I5R peaked at dawn for plants in winter samples, while it was not considered expressed for plants in summer samples (**Figures 2C**, **D**). The FS form for this region peaked between ZT04-05 for plants in both winter and summer samples (**Figure 2**).

*ScPRR37* I6R had a peak at ZT05 in winter samples, but at ZT07 in summer samples (**Figures 2E**, **F**). The corresponding FS isoform had a peak at ZT08 in both conditions. In turn, *ScPRR37* I7R had a peak at ZT07 in both winter and summer plants, but the corresponding FS isoform had a peak at ZT090 and ZT11 in winter and summer samples, respectively (**Figures 2G**, **H**). *ScPRR37* I3R and E3S were not detectable using HR RT-PCR (**Figures S7A**, **B**). Both the *ScPRR73* I6R and its FS isoform only had high levels in winter samples, with a peak at ZT07 (**Figures 2I**, **J**). The corresponding FS isoform had a peak at ZT10.

### Expressed AS Forms in the Different Source-Sink Sugarcane Organs

We extended the investigation to two other sugarcane organs, internodes 1 and 2 (I1) and internode 5 (I5). These internodes have different physiology: I1 has a high cellular and metabolic activity, whereas I5 is the first internode to actively accumulate sucrose. Only the plants harvested in the summer (9-month-old) had developed internodes that could be harvested. We only measured AS forms in genes *ScLHY* and *ScPRR37*, which were the homologs featuring the highest AS transcript expression in L1 (**Figure 2**). The main difference in expression levels between organs was observed in *ScPRR37* I6R, that had significantly higher levels in leaves during the day compared to the internodes (One-way ANOVA with post-hoc Tukey HSD test, ^*^p < 0.05, **Figure S8**).

**Figure 3:**
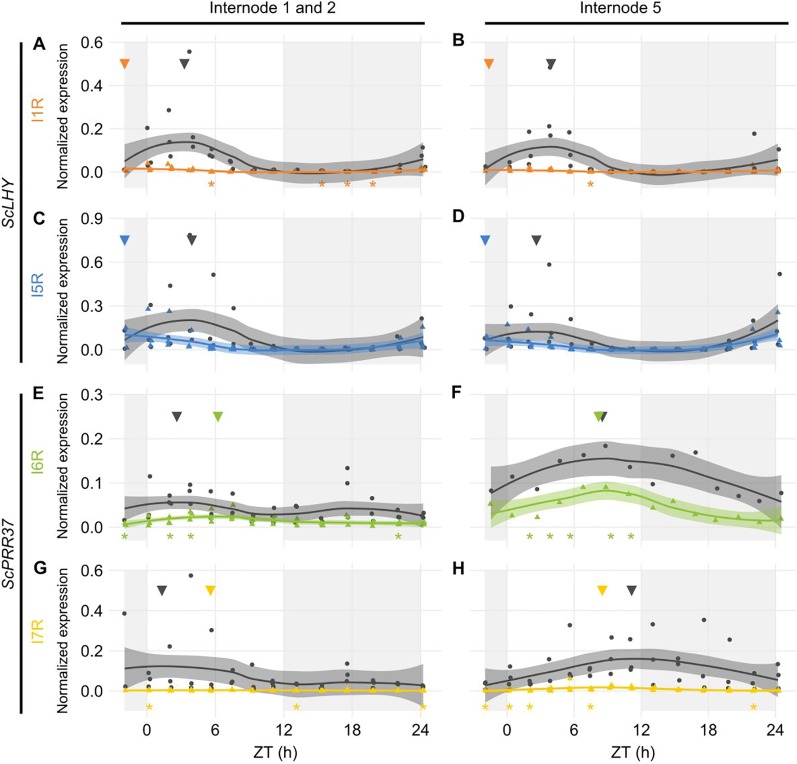
Diel expression profile of fully spliced and alternative spliced transcript isoforms in internodes. Biological replicates (circles and triangles) and their LOESS curve (continuous lines ± SE) of fully spliced (FS, black) and alternative spliced (AS, colored) transcript forms of sugarcane circadian clock genes during the summer harvest in internode 1 and 2 (left) and internode 5 (right). **(A**, **B)** *ScLHY* gene expression shows levels of I1R (orange) and **(C**, **D)** I5R (blue); **(E**, **F)** *ScPRR37* gene expression shows levels of I6R (green) and **(G**, **H)** I7R (yellow). Inverted triangles show the time of the maximum value of the LOESS curve. The light-gray boxes represent the night period. Statistical significance was analyzed by paired Student’s t-test, ^*^p < 0.05.

In both internodes, *ScLHY* showed detectable levels of both AS events observed in L1. *ScLHY* I1R-containing transcript levels were very low with a peak 2 h before dawn (ZT22), with the FS isoform peaking between ZT03-04 (**Figures 3A**, **B**). *ScLHY* I5R was also identified in both internodes, at higher levels than *ScLHY* I1R. The AS isoform peaked at ZT22, while the FS form peaked between ZT03-04 (**Figures 3C**, **D**). During the end of the night, *ScLHY* I1R levels were significantly higher in the leaves compared to the internodes (One-way ANOVA with post-hoc Tukey HSD test, ^*^p < 0.05, **Figure S8**).

The *ScPRR37* homolog featured variable levels of FS and AS isoforms when compared to both internodes. In I1, *ScPRR37* I6R and its FS isoform did not have a clear rhythm, with the I6R showing low levels of expression, and the FS isoform showing two peaks. In I5, *ScPRR37* I6R and its FS isoform had higher levels and a peak between ZT08-09 (**Figures 3E**, **F**). In both internodes, the I7R isoform was expressed at very low levels, but the FS isoform for that transcript region peaked at ZT01 in I1 and ZT11 in I5 (**Figures 3G**, **H**).

### Alternative Splicing Events Are Dependent on the Time of the Day and Temperature

**Figure 4:**
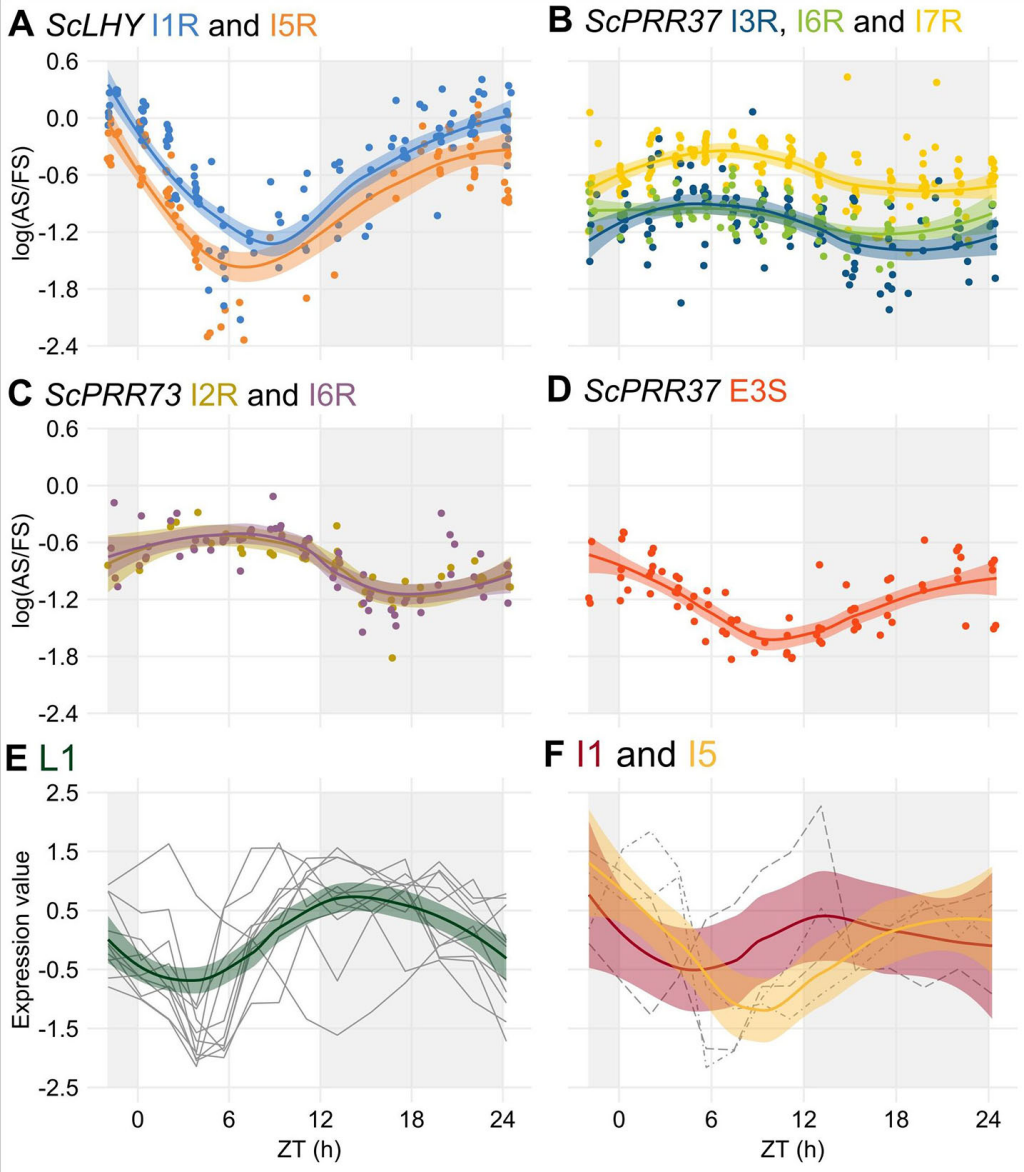
Alternative splicing is rhythmic in sugarcane in field conditions. **(A**–**D)** The logarithm of the ratio of the expression levels of an AS isoform to its FS isoform, annotated as the log(AS/FS), was plotted against the normalized time of the day (ZT). **(A)** *ScLHY* I1R (orange) and I5R (blue); *ScPRR37* I3R (dark blue), **(B)** I6R (green) and I7R (light yellow); **(C)** *ScPRR73* I2R (gold) and I6R (purple); and **(D)** *ScPRR37* E3S (red). **(E**–**F)** Normalized expression levels of rhythmic splicing-related transcripts taken from oligo array data (Dantas et al., 2019) in **(E)** leaves +1 (L1, green), and internodes 1 and 2 (I1, red) and **(F)** internode 5 (I5, yellow). Individual expression profiles were drawn in gray. LOESS regression was used to draw the trends in the data in all panels (continuous line ± SE). The light-gray boxes represent the night period.

After the identification of rhythms in FS and AS transcripts, we tried to identify rhythms in their relative levels by examining the log of the splicing ratio of the AS to the FS transcripts [log (AS/FS)] from the HR RT-PCR data. *ScLHY*, *ScPRR37,* and *ScPRR73* showed evidence of splicing rhythms (**Figure 4**), but only *ScLHY* had more than a 10-fold difference between the maximum and the minimum log(AS/FS) (77-fold) (**Figure 4A**). The AS : FS ratios of all the time-point samples and organs were grouped, as they showed similar rhythmic patterns. In general, all AS events of a gene showed a similar rhythmic pattern. The only exception was E3S in *ScPRR37* (**Figure 4D**), that had a different phase from the other AS events found in *ScPRR37* (**Figure 4B**). The AS events observed in *ScLHY* and E3S in *ScPRR37* had a peak at the end of the night, between ZT20 and ZT24, and a trough between ZT06 and ZT08. In contrast, *ScPRR73* AS events and the remaining *ScPRR37* AS events had a peak between ZT05 and ZT06, and a trough between ZT16 and ZT18.

The rhythmic changes in the log(AS/FS) values could be explained by changes in the expression of putative regulatory genes, such as splicing factors or spliceosomal protein genes. In a previous work, we have identified 6,705 rhythmic transcripts in L1, 3,755 in I1 and 3,242 in I5 in field-grown sugarcane (Dantas et al., 2019). Fourteen spliceosome-related transcripts in the oligo array were expressed in all three organs (**Table S2**): 9 transcripts were rhythmic only in L1, one was rhythmic in L1 and I1, one was rhythmic in L1 and I5, and one was rhythmic in I1 and I5 (**Table S3**). In L1, 9 of the 11 rhythmic transcripts peaked between ZT09 and ZT13, with a trough between ZT03 and ZT5 (**Figure 4E**). In the internodes, most of the transcripts peaked at ZT00 (**Figure 4F**).

**Figure 5:**
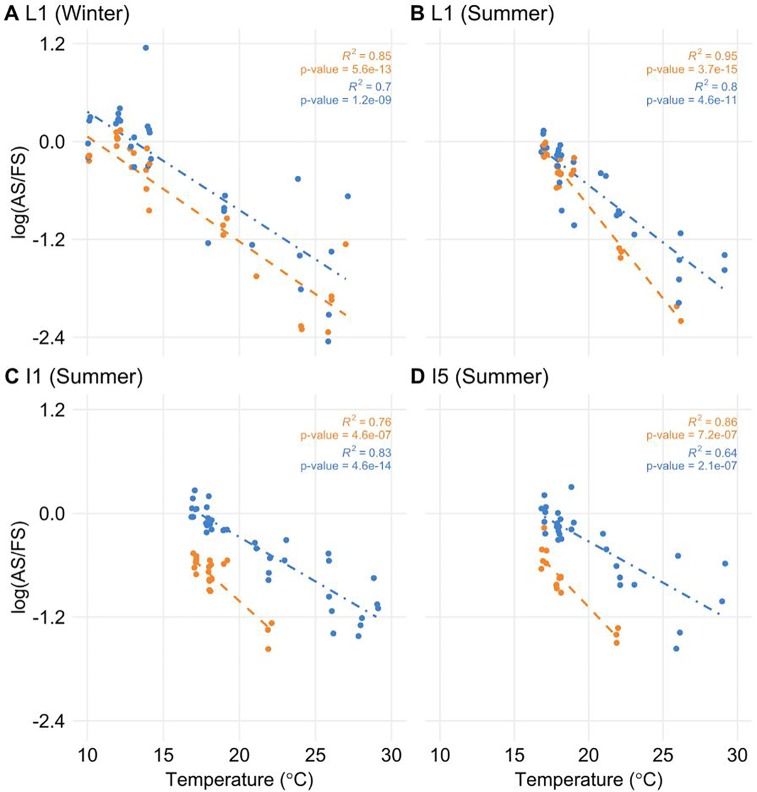
The proportion of alternative spliced and fully spliced forms has a negative correlation with ambient temperature. The logarithm of the ratio of the expression levels of AS and FS isoforms, annotated as the log(AS/FS), was plotted against the ambient temperature for *ScLHY* I1R (orange) and I5R (blue). Regression lines were added for each group of ratios. R^2^ and *P-value* were calculated for each regression. Negative correlations were significant in leaf +1 (L1) in the **(A)** winter (4-month-old plants), and **(B)** summer, **(C)** internode 1 and 2 (I1), and **(D)** internode 5 (I5), both in the summer (9-month-old plants).

To test if the temperature was a factor in the regulation of AS, we correlated temperature information with log(AS/FS) values. Only *ScLHY* AS events showed a significant negative correlation (**Figure 5**). The negative correlation was found in both *ScLHY* AS events, at both harvests/seasons, and all organs. This suggests that the AS regulation of *ScLHY* genes are temperature-dependent.

## Discussion

In this paper, pioneer information on AS events in the sugarcane circadian clock core genes *ScLHY*, *ScPRR37*, *ScPRR73*, *ScPRR95*, and *ScTOC1* in field-grown sugarcane plants are presented (**Figure 1**). As for *Arabidopsis* and barley in previous studies, AS is widespread among circadian clock genes (Filichkin et al., 2010; Filichkin and Mockler, 2012; James et al., 2012a; Calixto et al., 2016). The circadian clock homolog *CCA1/LHY* in rice also displays the conserved I1R AS event, suggesting conserved patterns in AS events between the two species (Filichkin et al., 2015a). In barley, AS events were described for the homologs *HvLHY*, *HvPRR37*, *HvPRR73,* and *HvGI*. There are conserved AS isoforms expressed in both *Arabidopsis* and barley for *HvLHY* and *HvPRR37* (Calixto et al., 2016). The conservation of expression of AS forms across different plant species highlights the role that AS plays in gene expression of circadian clock genes. Our data in sugarcane identified at least one AS event in each of the clock gene homologs analyzed. Given the ploidy of sugarcane, it was necessary to demonstrate that the alternative transcript sequences did not originate from haplotypes but were *bona fide* AS events. By examination of existing sequenced genomic data, we conclude that they are AS events. The most frequent AS event in the sugarcane circadian clock genes was intron retention (IR), which was detected in all five genes. The presence of transcripts containing retained introns might also be an indication of partially spliced transcripts but the other introns in these genes were efficiently removed. The presence of retained introns in transcript isoforms can lead to post-transcriptional regulation of gene expression (Reddy et al., 2013). Since retained introns usually insert premature termination codons (PTCs) in the transcript, they could be substrates for degradation *via* the nonsense-mediated decay (NMD) pathway. However, in plants, transcripts with detained introns appear to avoid NMD since their abundance is unaffected in NMD mutants (Kalyna et al., 2012; Marquez et al., 2012). Furthermore, such transcripts have been shown to remain in the nucleus and thus avoid the NMD machinery (Göhring et al., 2014). Nuclear intron detention is now recognized as an important post-transcriptional regulatory mechanism (Jacob and Smith, 2017). Intron retention transcripts with PTCs can also potentially give rise to C-terminally truncated and dysfunctional proteins (Seo et al., 2011; Mastrangelo et al., 2012; Reddy et al., 2013). Other AS events in sugarcane circadian clock genes that were in frame and did not insert a PTC in the transcript were the alternative 5′ splice site in ScPRR37 exon 3 (Alt 5′ss E3) and the *ScPRR73* alternative splice site between exons 4 and 5: Alt 5′ss E4 (‑104) and Alt 3′ ss E5 (‑142) (**Table S2**). In both cases, there is the removal of nucleotides from the coding sequence. *ScPRR37* Alt 5′ss E3 removes 30 nucleotides (10 amino acids) from the PRR domain. The resulting sequence is likely to be translated into a defective protein. In *ScPRR73*, the combination of Alt 5′ss E4 (-104) and Alt 3′ ss E5 (‑142) removes 246 nucleotides (82 amino acids) from the coding sequence but still leaves an ORF. However, it is possible that the loss of sequence could affect the normal function of the *ScPRR73* CTT domain. Therefore, the AS events identified in the sugarcane core circadian clock genes either produce transcripts that are likely to be kept in the nucleus and degraded or that are translated into incomplete proteins that are likely to be functionally defective. Thus, AS has an important role in regulating expression and production of core clock proteins. This might have a direct impact on the clock-dependent plant metabolism and physiology.

The levels of alternative transcript isoforms can vary under stress conditions (Staiger and Brown, 2013b; Shang et al., 2017; Calixto et al., 2018), at specific developmental stages (Szakonyi and Duque, 2018) or in different cell tissues (Shen et al., 2014; Thatcher et al., 2014). In previous work using microarrays, we found that rhythmic expression at the gene level was very organ-specific in sugarcane (Dantas et al., 2019). Our data shows differences in the AS in leaf and internodes at the transcript level. In L1, circadian clock transcripts undergo AS at higher relative levels than in I1 and I5 at the end of the night (**Figures 2** and **3**). In addition, the splicing ratios, log(AS/FS), had rhythms in *ScLHY*, *ScPRR37*, and *ScPRR73*. In *ScPRR37*, the intron retention events peaked in the middle of the day, while the exon skipping event peaked at the end of the night, suggesting that the temporal regulation of these two types of AS events are independent of each other (**Figures 4B**–**D**). Although their expression profiles differ, the consequences of *ScPRR37* I3R and E3S events are likely to be similar. I3R introduces PTCs after exon 3 and E3S removes exon 3 (part of the PRR domain). *Arabidopsis PRR7* also has two mutually exclusive AS events in a similar region of the gene: retention of intron 3 (I3R) and skipping of exon 4 (E4S), both of which give nonproductive mRNAs (James et al., 2012a). The switch from intron retention (mainly during the day) to exon skipping (mainly during the night) in *ScPRR37* may reflect rhythmic changes in specific splicing factors. The splicing ratio rhythms of *ScLHY* and *ScPRR73* showed the same pattern, irrespective of the sampling season or organ (**Figures 4A**–**C**). This means that these splicing ratios are not organ-specific and are environmentally and circadian clock-regulated in order to have the same distribution during the day and the night regardless of their duration. To try and relate the rhythmic changes in splicing ratios of clock genes to the expression of splicing factor or spliceosomal protein genes, we examined the expression of the splicing-related genes that were rhythmic in L1 in our previous microarray study (**Figure 4E**) (Dantas et al., 2019). The gene-level expression of the majority of these genes peak around dusk (between ZT11 and ZT13), which does not coincide with the peaks in splicing ratios observed in circadian clock genes. The list of splicing-related genes in the microarray analysis was not extensive (**Table S1**) and many splicing factors are alternatively spliced to regulate the level of productive, protein-coding transcripts (Reddy et al., 2013; Staiger and Brown, 2013a). Transcript level RNA-seq will be required to more accurately measure protein-coding transcripts of splicing factors to identify candidate regulators of AS of the core clock genes analyzed here.

Another interesting observation is the noticeable variation in the AS transcripts across the different organs analyzed (**Figure 3**). The differences in transcript expression between source and sink tissues might reflect their metabolical differences. While L1 is a fully photosynthetically active leaf in sugarcane, therefore undergoing photosynthesis, both internodes sink in the assimilated carbon for different purposes: cell division and elongation in I1 and sucrose storage in I5. In *Arabidopsis*, sucrose has been shown to decrease *PRR7* levels, which decrease *ScLHY* levels as a consequence (Haydon et al., 2013; Frank et al., 2018). Thus, differences in *ScPRR37* I6R and *ScLHY* I1R levels (**Figures 3** and **S8**) could be due to the sucrose that is stored in the internodes. In turn, these differences in the levels of circadian clock genes might affect circadian clock outputs. We have found that transcriptional rhythms are very organ-specific in sugarcane (Dantas et al., 2019).

In *Arabidopsis*, the circadian clock is associated with photosynthesis (Dodd et al., 2009), cell division (Fung-Uceda et al., 2018), and sugar accumulation (Graf et al., 2010; Graf and Smith, 2011; Ko et al., 2016). Taken together, these data suggest that the circadian clock might regulate the different sugarcane organs in distinctive ways. It is already known that metabolites can feedback to regulate the circadian clock, as data show in *Arabidopsis* that photosynthetic sugars regulate clock functioning (Haydon et al., 2013). Considering that in *Arabidopsis* different tissues are enriched with different levels of circadian clock transcripts (Endo et al., 2014), a similar phenomenon might occur in sugarcane and explain the different organ transcript expressions and profiles, as well as helping to keep each organ different metabolic and physiologic profiles.

We found that the splicing ratios from *ScLHY* AS transcripts are negatively correlated with temperature in all organs. Previous studies on the presence of AS in circadian clock genes in *Arabidopsis* and barley revealed that, under controlled conditions, the AS status of circadian clock genes is regulated by temperature, especially *LHY* and its paralog *CCA1* (James et al., 2012a;  James et al., 2012b; Park et al., 2012; Seo et al., 2012; Kwon et al., 2014; Filichkin et al., 2015a; Calixto et al., 2016; Marshall et al., 2016; Calixto et al., 2018). Lower temperatures led to increased abundance of alternative non-productive forms of *LHY*, *PRR7* and *PRR5* in *Arabidopsis* (James et al., 2012a). This could affect the expression of fully spliced transcripts of the circadian clock genes, promoting a functional modulation in the circadian clock central oscillator, which might be reflected by altered temporal control of the clock outputs. In *Arabidopsis*, cold temperatures reduced the amplitude of *CCA1*/*LHY*, as well as disrupted the circadian clock function (Bieniawska et al., 2008). Considering the natural environment context, where plants like sugarcane face fluctuations in temperature on a daily and yearly basis, the continuous temperature-regulation of AS of the circadian clock network could have a more profound impact on metabolism and, ultimately, on crop yield.

The data in our work shows that from winter to summer, as the temperature increases (**Figure S1B**), the expression of alternative forms of the circadian clock genes decreases, noticeably for *ScLHY* (**Figures 2A**–**D**). In *Arabidopsis*, there is evidence linking the circadian clock with sugar accumulation as starch through *CCA1* and *LHY* (Ni et al., 2009; Miller et al., 2012; Ng et al., 2014) and in field-grown maize, a C4 plant like sugarcane, two *CCA1* homologs are associated to photosynthesis and, therefore, sugar accumulation (Ko et al., 2016). All this evidence allows us to speculate that there might be differences in the sugar accumulation by the field-grown sugarcane from winter to summer, but a metabolomic analysis focused on sucrose and hexoses content would be necessary to bring evidence to support such speculation.

Recent studies featuring experiments conducted in field conditions in *Arabidopsis*, rice and tomato highlight the differences in gene expression, circadian regulation and plant metabolism compared to experiments conducted inside growth chambers (Annunziata et al., 2017; Annunziata et al., 2018; Izawa et al., 2011b; Higashi et al., 2016; Shalit-Kaneh et al., 2018). Because AS has an impact on the regulation of gene expression, which impacts circadian regulation and plant metabolism, it is important to start investigating the dynamic adjustment of AS in response to a fluctuating environment. RNA-seq data from time-series experiments revealed that the AS status of *Arabidopsis* transcriptome is widely responsive to changes in temperature (Calixto et al., 2018). By progressively lowering temperature, rapid changes in the spliced forms of transcripts were detected, which suggests that AS might also act to regulate low-temperature responses and how plants tolerate such stress (Calixto et al., 2018). The *Arabidopsis* circadian clock also acts in regulating plant abiotic stress tolerance (Grundy et al., 2015). This also suggests that both AS and the circadian clock might act in synergy to help plants to cope with temperature changes in both the short and long term. In the field, this regulation might be even more important, due to the unexpected fluctuations in light, temperature, and humidity in which plants are exposed.

Our data show that AS occurs in sugarcane circadian clock genes and that the different transcript isoforms show a dynamic expression profile in sugarcane grown under field conditions. Furthermore, *ScLHY* AS regulation correlates with temperature in sugarcane circadian clock genes. Thus, the changes in expression of alternative isoforms of *ScLHY* transcripts observed across winter and summer might illustrate the combined effect of both the circadian clock and AS regulation and AS in *ScLHY* might be a key mechanism that allows the continuous dynamic adjustment of the circadian clock by temperature in sugarcane. It is important to start further studies on the impact of the seasonal variation on the AS isoforms of the circadian clock gene expression and, ultimately, sugarcane metabolism and yield.

## Data Availability Statement

All datasets generated for this study are included in GitHub (https://github.com/LabHotta/AlternativeSplicing) and archived on Zenodo (http://doi.org/10.5281/zenodo.3509232).

## Author Contributions

CH and JB designed this research. LD and CH harvested the biological material and carried out BLAST analyses. LD processed all samples and carried out cloning and HR RT-PCRs. LD, CC, JB, and CH participated in the interpretation of genomic annotation and HR RT-PCR data. MD contributed with cloning. MC contributed with the plants and space for the field experiment. LD and CH drafted the manuscript. All authors participated in its correction and have read and approved the final manuscript. CH and JB acquired the funding.

## Funding

The present study was supported by the São Paulo Research Foundation (FAPESP) [grant nos. 11/00818-8 and 15/06260-0; BIOEN Program], and by the Serrapilheira Institute (grant no. Serra-1708-16001). LD was supported by FAPESP scholarships [grants 11/08897-4 and 15/10220-3]. CC and JB were supported by funding from the Biotechnology and Biological Sciences Research Council (BBSRC) [BB/K006568/1 and BB/N022807/1] and the Scottish Government Rural and Environment Science and Analytical Services division (RESAS) [to JB].

## Conflict of Interest

The authors declare that the research was conducted in the absence of any commercial or financial relationships that could be construed as a potential conflict of interest.
