# Supplementary material for: Alternative Splicing of Circadian Clock Genes Correlates With Temperature in Field-Grown Sugarcane
Source: Front Plant Sci. 2019 Dec 23;10:1614. doi: 10.3389/fpls.2019.01614 (PMC6936171; doi:10.3389/fpls.2019.01614)
Supplement: Supplementary file 1 [file DataSheet_1.docx]

**Table S1 – List of sugarcane gene-specific primers and their sequences.** Primers in bold indicate forward (FW) sequences, as well as the primers bearing the 6-FAM tag used in the HR RT-PCR assays. The other sequences indicate reverse (RV) sequences. Normalizer primers are highlighted in green.

| **Gene** | **Sequence (5' → 3')** |
| --- | --- |
| *ScGAPDH* | **CACGGCCACTGGAAGCA** |
|  | TCCTCAGGGTTCCTGATGCC |
| *ScPP2AA2* | **GCGCCTTAACTCCATCAGAA** |
|  | GCTCTACACCTCCAACATAAGG |
| *ScLHY* | **CTGTTGCACATTTCCCTTCTG** |
|  | TTTGAGCGTGACTTCTGATCT |
|  | **ACCTACCACAGGAGCTGATA** |
|  | GATAAATGCTTGTGGCGTTCTT |
| *ScTOC1* | **ATTCTTCTCTGCGACGGCGA** |
|  | TAGGCCTATCGTCTGTCTCGT |
| *Sc*PRR37 | **GGAATGACAATCTGAGGAGCAG** |
|  | AGTTCATTCTTACGTATAGGCTTGG |
|  | **TGTGGAGATGGTGCCAATAC** |
|  | GCAACATCACCCTGTCCTTAT |
|  | GAGCAACTCAGGCAGTAACA |
|  | GCATAGCTTGCTTTACGAACTG |
| *ScPRR73* | **GAGAGGTTCCTACCTGTGAAGA** |
|  | CTGTGTCTGGATGCCACTTT |
|  | **GTGACGAGTATGAGAACGACAG** |
|  | GAGTTCCTAGGAGCACCTATTTC |
|  | **CAAGATGCCCGATGGAAATG** |
|  | GACGTTACTCTCTCCCTGTTAG |
|  | **GGGTGTCTCAGTTGGTCATAAT** |
|  | TGAACCTTGCGCTGCTT |
| *Sc*PRR95 | **AGTGGACTTCCTTGTGAAACC** |
|  | GAACCAGCCACTTCATCTCTAC |
|  | **ATTCTTCGGGAATCAGGAGAAC** |
|  | CAGTTTCTGCTTCTGTTGTTGTAG |

**TableS2 – SAS number, GeneID, and ProbeID of genes associated with the Spliceosome in the Sugarcane array (Dantas et al., 2019).**

| **Gene** | **SAS** | **Gene ID** | **Probe ID** |
| --- | --- | --- | --- |
| *ABH1* | SCCCCL4004G07.g | TC136430 | GS1_SS_06377_20908 |
| *CDC5* | SCJFRZ2005G12.g | TC116361 | GS1_SS_18244_14104 |
| *LSM8* | SCCCAM2004E11.g | TC126680 | GS1_SS_22616_12065 |
| *LSM36B* | SCCCLR2002D01.g | TC124904 | GS1_SS_10307_02774 |
|  | SCEZAM1081G05.g | TC132981 | GS1_SS_24478_05144 |
| *Prp3* | SCEPAM1022H06.g | TC123686 | GS1_SS_04144_21793 |
| *RCF1* | SCVPAM1059D07.g | TC133378 | GS1_SS_16968_10290 |
| *SC35* | SCSFSB1101H12.g | TC113063 | GS1_SS_22263_08222 |
| *SmD2* | SCACLR2014D10.g | TC117969 | GS1_SS_07124_11310 |
| *SmF* | SCEPLB1043F04.g | TC140865 | GS1_SS_02829_12937 |
| *SR34* | SCCCLR2C03A06.g | TC114613 | GS1_SS_12661_19184 |
| *STA1* | SCUTSB1075G02.g | TC130590 | GS1_SS_17461_08595 |

**Table S3 – Spliceosome-related transcripts showing rhythmicity in field conditions according to Dantas et al. (2019).** Sugarcane Assembled Sequences (SAS) were taken from the SUCEST-FUN database (http://sucest-fun.org).

| **Organ** | **Gene** | **Sugarcane Assembled Sequences (SAS)** | **Arabidopsis homologs** | **Phase (ZT)** |
| --- | --- | --- | --- | --- |
| Internodes 1 and 2 | *Prp3* | SCEPAM1022H06.g | At1g28060 | 0 |
|  | *LSM8* | SCCCAM2004E11.g | At1g65700 | 11 |
|  |  |  |  |  |
| Internode 5 | *ABH1* | SCCCCL4004G07.g | At2g13540 | 0 |
|  | *Prp3* | SCEPAM1022H06.g | At1g28060 | 0 |
|  |  |  |  |  |
| Leaf +1 | *RCF1* | SCVPAM1059D07.g | At1g20920 | 2 |
|  | *LSM8* | SCCCAM2004E11.g | At1g65700 | 9 |
|  | *SmD2* | SCACLR2014D10.g | At2g47640 | 9 |
|  | *CDC5* | SCJFRZ2005G12.g | At1g09770 | 11 |
|  | *LSM36B* | SCCCLR2002D01.g | At3g59810 | 11 |
|  |  | SCEZAM1081G05.g | At5g27720 | 11 |
|  | *SR34* | SCCCLR2C03A06.g | At1g02840 | 11 |
|  | *ABH1* | SCCCCL4004G07.g | At2g13540 | 13 |
|  | *SC35* | SCSFSB1101H12.g | At5g64200 | 13 |
|  | *SmF* | SCEPLB1043F04.g | At4g30220 | 13 |
|  | *STA1* | SCUTSB1075G02.g | At4g03430 | 22 |
