# Supplementary material for: Alternative Splicing of Circadian Clock Genes Correlates With Temperature in Field-Grown Sugarcane
Source: Front Plant Sci. 2019 Dec 23;10:1614. doi: 10.3389/fpls.2019.01614 (PMC6936171; doi:10.3389/fpls.2019.01614)
Supplement: Supplementary file 2 [file DataSheet_2.docx]

**Supplemental Figure 1 – Field design and environmental conditions.** **(A)** The experimental field consisted of 8 plots, that were surrounded by two lines of sugarcane. There were 4 lines per plot, each line 5 m long and filled with 20 tillers of commercial sugarcane (variety SP80-3280). The border lines (dashed yellow lines) were not used in our experiment in order to avoid border effects. Plants from the two central lines of two plots were randomly picked and randomly assigned to three pools. Temperature **(B)** and light intensity **(C)** during the summer sampling (red continuous line) and the winter sampling (dashed blue line) were obtained from a nearby weather station. The time points were normalized for a 12h photoperiod. The light-gray boxes represent the night period.

**Supplemental Figure 2 – Alignment of two different regions of ScLHY from different genomic sequences.** Sequences from different sugarcane genome projects that were identified as *ScLHY* were aligned in order to compare the regions containing the I1R and I5R AS events in different haplotypes. The regions containing (**A**) the intron 1 (orange) and (**B**) intron 5 (blue) were highlighted to show that all the sequenced haplotypes have the same gene structure. Sequences are from *Sorghum bicolor* (SB) genome (McCormick et al., 2018), *S. spontaneum* (SS) (Zhang et al., 2018), *Saccharum* hybrid (SP80-3280): SHs (Souza et al., 2019), SHr (Matiello et al., 2017), and *Saccharum* hybrid (R570) (SHg) (Garsmeur et al., 2018).

**Supplemental Figure 3 – Alignment of three different regions of *ScPRR37* from different genomic sequences.** Sequences from different sugarcane genome projects that were identified as *ScPRR37* were aligned in order to compare the regions containing the I3R, I6R, and I7R AS events in different haplotypes. The regions containing (**A**) the intron 3 (dark blue), (**B**) intron 6 (green) and (**C**) intron 7 (yellow) were highlighted to show that all the sequenced haplotypes have the same gene structure. Sequences are from *S. spontaneum* (SS) (Zhang et al., 2018), *Saccharum* hybrid (SP80-3280): SHs (Souza et al., 2019), SHr (Matiello et al., 2017), and *Saccharum* hybrid (R570) (SHg) (Garsmeur et al., 2018).

**Supplemental Figure 4 – Alignment of two different regions of *ScPRR73* from different genomic sequences.** Sequences from different sugarcane genome projects that were identified as *ScPRR73* were aligned in order to compare the regions containing the I2R and I6R AS events in different haplotypes. The regions containing (**A**) the intron 2 (gold) and (**B**) intron 6 (purple) were highlighted to show that all the sequenced haplotypes have the same gene structure. Sequences are from *S. spontaneum* (SS) (Zhang et al., 2018), *Saccharum* hybrid (SP80-3280): SHs (Souza et al., 2019), SHr (Matiello et al., 2017), and *Saccharum* hybrid (R570) (SHg) (Garsmeur et al., 2018).

**Supplemental Figure 5 – Alignment of two different regions of *ScPRR95* from different genomic sequences.** Sequences from different sugarcane genome projects that were identified as *ScPRR95* were aligned in order to compare the regions containing the I3R and I7R AS events in different haplotypes. The regions containing (**A**) the intron 3 (pink) and (**B**) intron 7 (dark green) were highlighted to show that all the sequenced haplotypes have the same gene structure. Sequences are from *S. spontaneum* (SS) (Zhang et al., 2018), *Saccharum* hybrid (SP80-3280): SHs (Souza et al., 2019), SHr (Matiello et al., 2017), and *Saccharum* hybrid (R570) (SHg) (Garsmeur et al., 2018).

**Supplemental Figure 6 – Alignment of two different regions of *ScTOC1* from different genomic sequences.** Sequences from different sugarcane genome projects that were identified as *ScTOC1* were aligned in order to compare the regions containing the I1R and I3R AS events in different haplotypes. The regions containing (**A**) the intron 1 (light blue) and (**B**) intron 3 (red) were highlighted to show that all the sequenced haplotypes have the same gene structure. Sequences are from *S. spontaneum* (SS) (Zhang et al., 2018), *Saccharum* hybrid (SP80-3280): SHs (Souza et al., 2019), SHr (Matiello et al., 2017), and *Saccharum* hybrid (R570) (SHg) (Garsmeur et al., 2018).

**Supplemental Figure 7 –** **Diel expression profile of fully spliced and alternative transcript isoforms in different seasons.** Biological replicates (circles and triangles) and their LOESS curve (continuous lines ± SE) of fully spliced (FS, black) and alternative transcript forms (AS, color) for the winter samples (4-months old plants, left) the summer samples (9-months old plants, right). These AS forms, though detected by HR RT-PCR, were not highly expressed. *ScPRR37* gene expression shows levels of I3R (orange) and E3S (dark blue) (**A-B**); *ScPRR73* gene expression shows levels of I2R (gold) (**C-D**); *ScPRR95* gene expression shows levels of I3R (pink) and Alt 5’ss E5 (teal) (**E-F**), and I7R (dark green) (**G-H**). *ScTOC1* gene expression shows levels of I1R/E23S (cyan) (**I-J**). Inverted triangles show the time of the maximum value of the LOESS curve. The light-gray boxes represent the night period. Statistical significance was analyzed by paired Student’s t-test, * p < 0.05 (C-D, G-J) or by One-way ANOVA with post-hoc Tukey HSD test, * p < 0.05 (A-B, E-F), each color corresponding to a test of an AS form against the FS form.

**Supplemental Figure 8 – Diel expression profile of fully spliced and alternative spliced transcript isoforms in leaves and internodes.** Biological replicates (circles and triangles) and their LOESS curve (continuous lines ± SE) of fully spliced (left column) and alternative spliced (right column) transcript forms of sugarcane circadian clock genes during the summer harvest in leaves (green), internode 1 and 2 (red) and internode 5 (yellow). **(A, B)** *ScLHY* gene expression shows levels of I1R and **(C, D)** I5R; **(E, F)** *ScPRR37* gene expression shows levels of I6R and **(G, H)** I7R. The light-gray boxes represent the night period. Statistical significance was analyzed by One-way ANOVA with post-hoc Tukey HSD test, * p < 0.05 when comparing L1 against I1, # p < 0.05 when comparing L1 against I5, and † p < 0.05 when comparing I1 against I5.
