# Supplementary figures and images for: Alternative Splicing of Circadian Clock Genes Correlates With Temperature in Field-Grown Sugarcane
Source: Front Plant Sci. 2019 Dec 23;10:1614. doi: 10.3389/fpls.2019.01614 (PMC6936171; doi:10.3389/fpls.2019.01614)

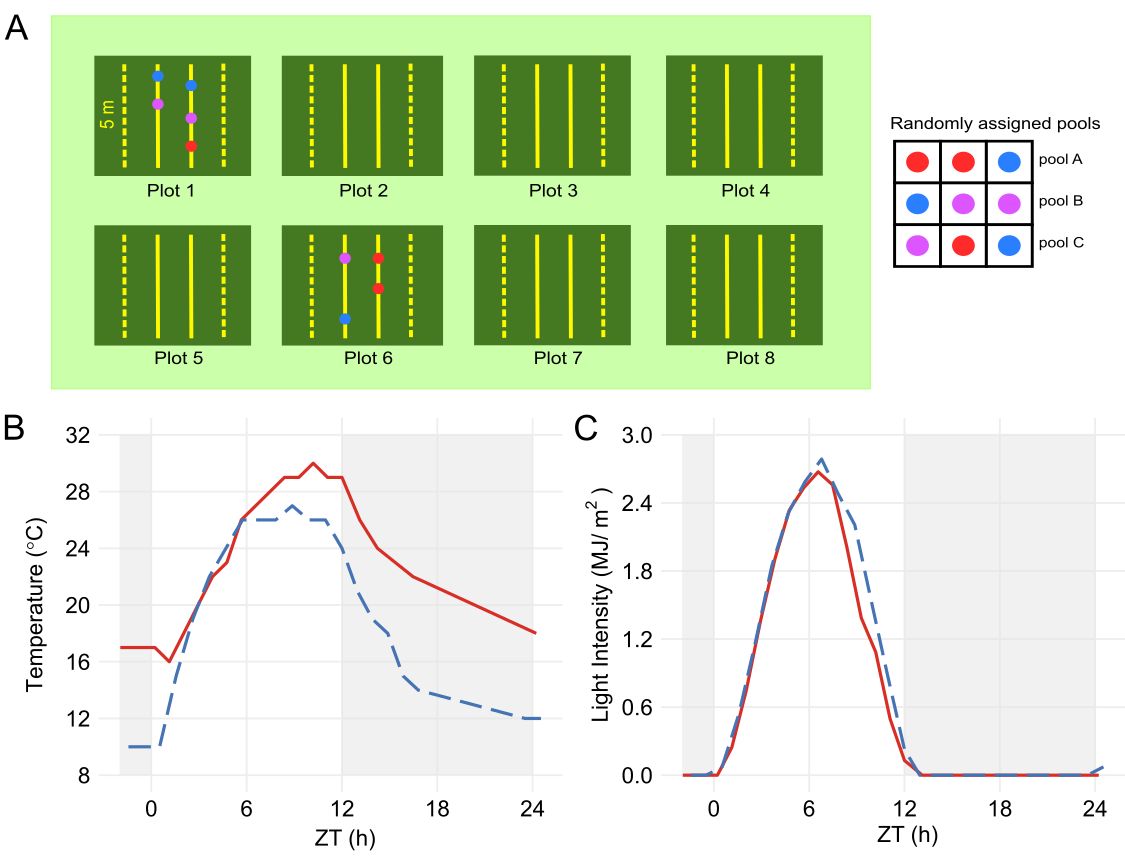

Supplement: Supplementary file 3 [file Image_1.jpeg]

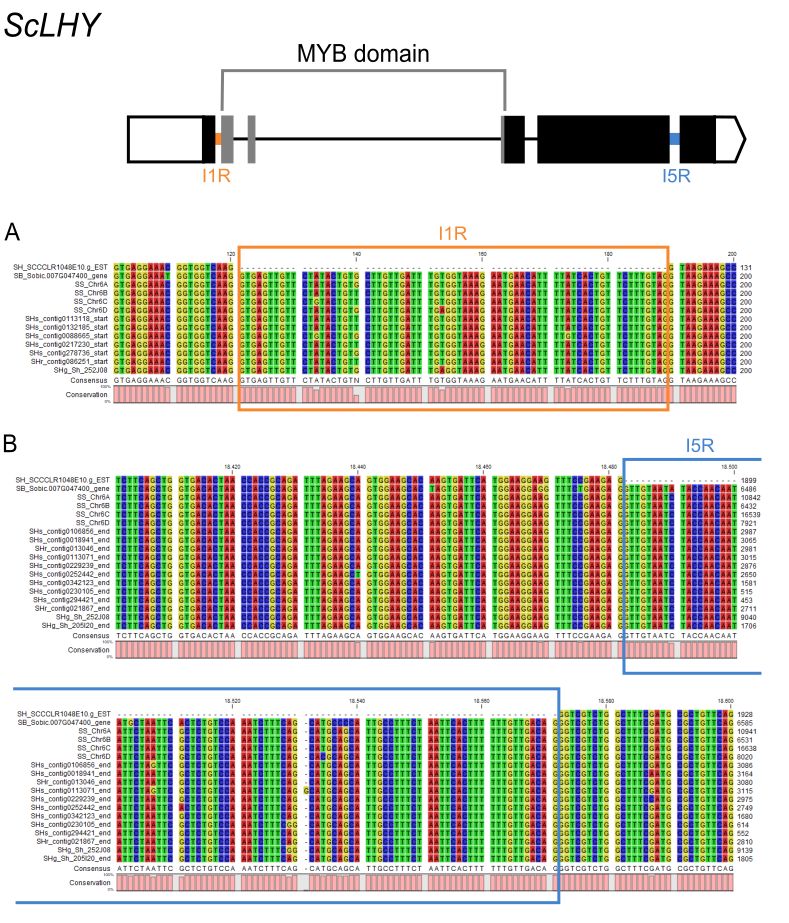

Supplement: Supplementary file 4 [file Image_2.jpeg]

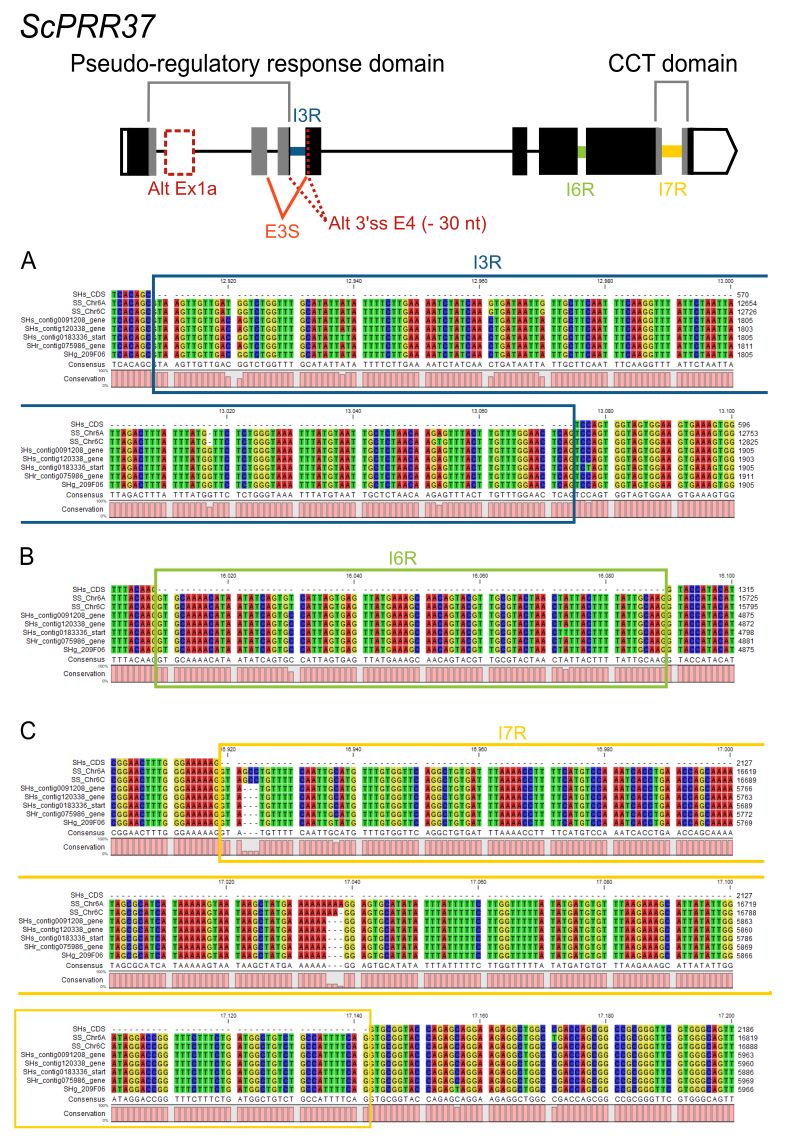

Supplement: Supplementary file 5 [file Image_3.jpeg]

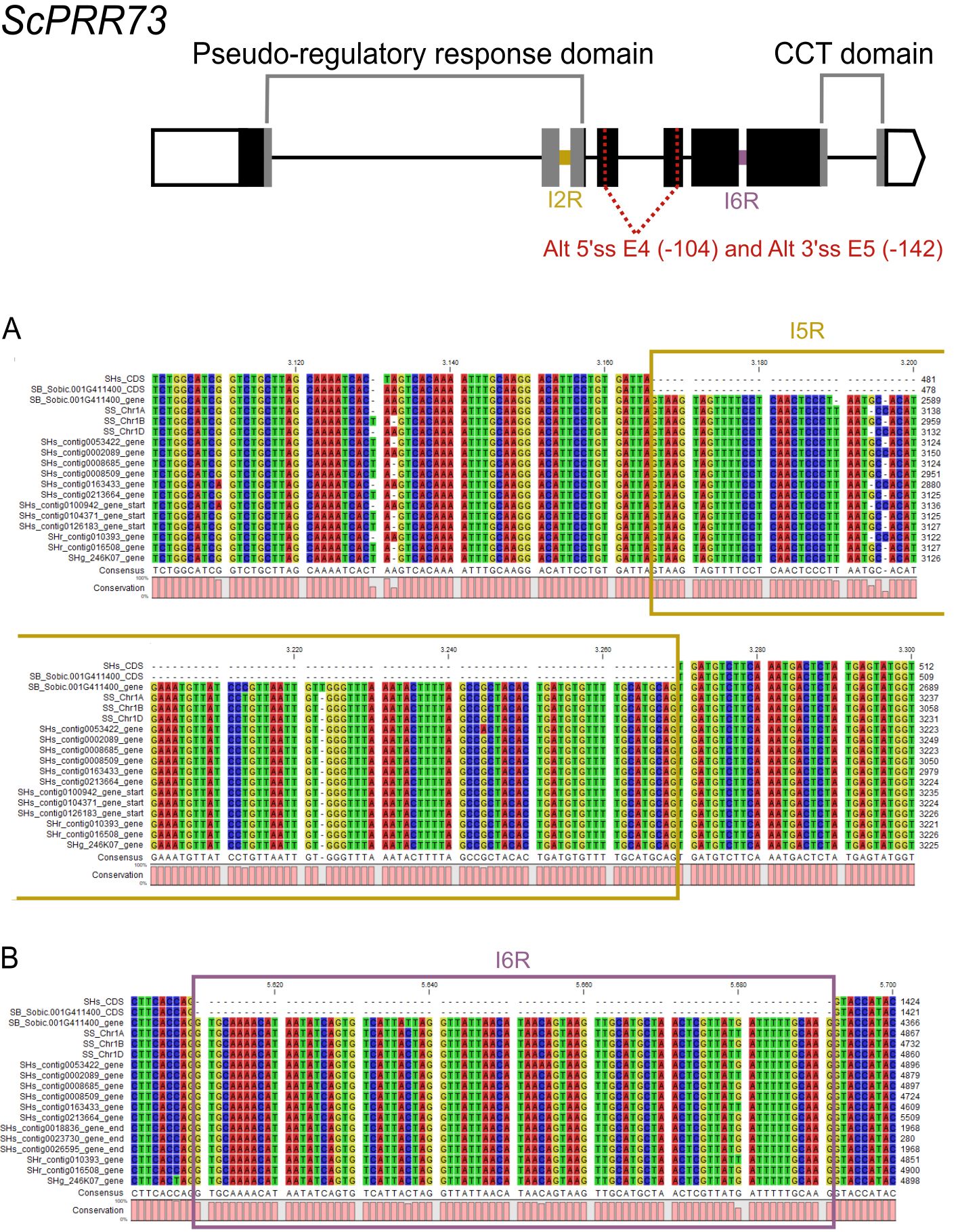

Supplement: Supplementary file 6 [file Image_4.jpeg]

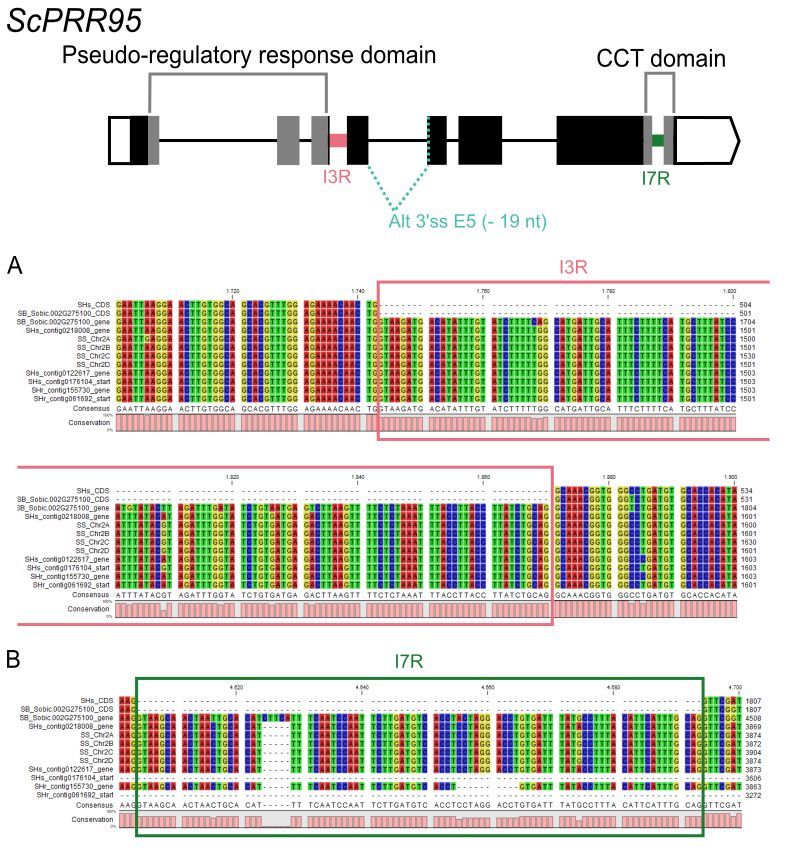

Supplement: Supplementary file 7 [file Image_5.jpeg]

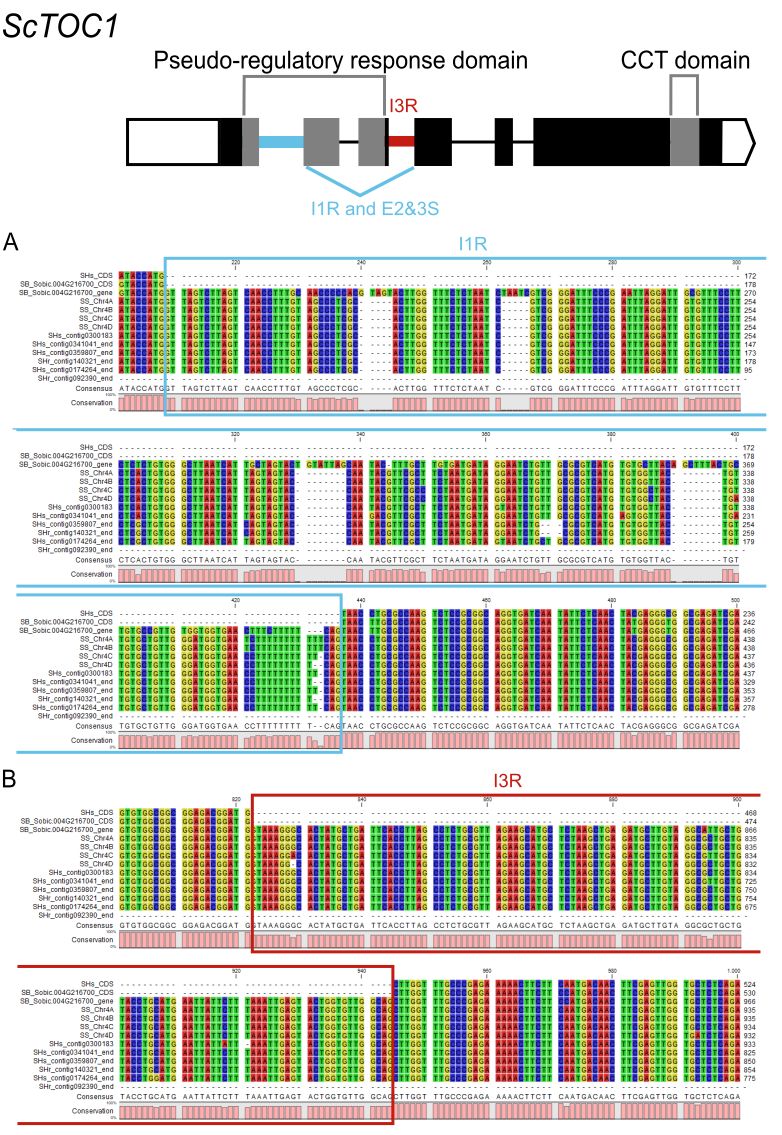

Supplement: Supplementary file 8 [file Image_6.jpeg]

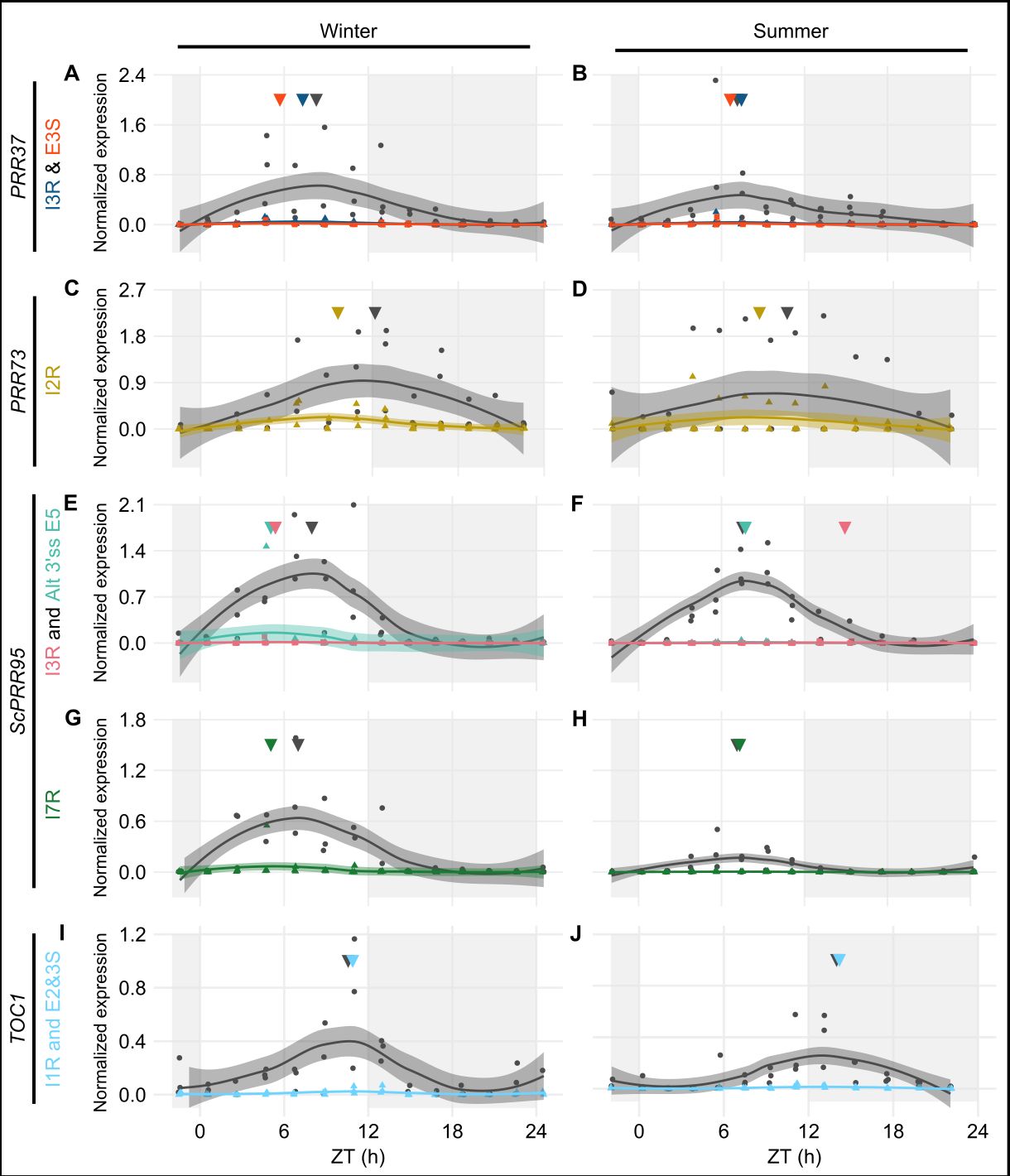

Supplement: Supplementary file 9 [file Image_7.jpeg]

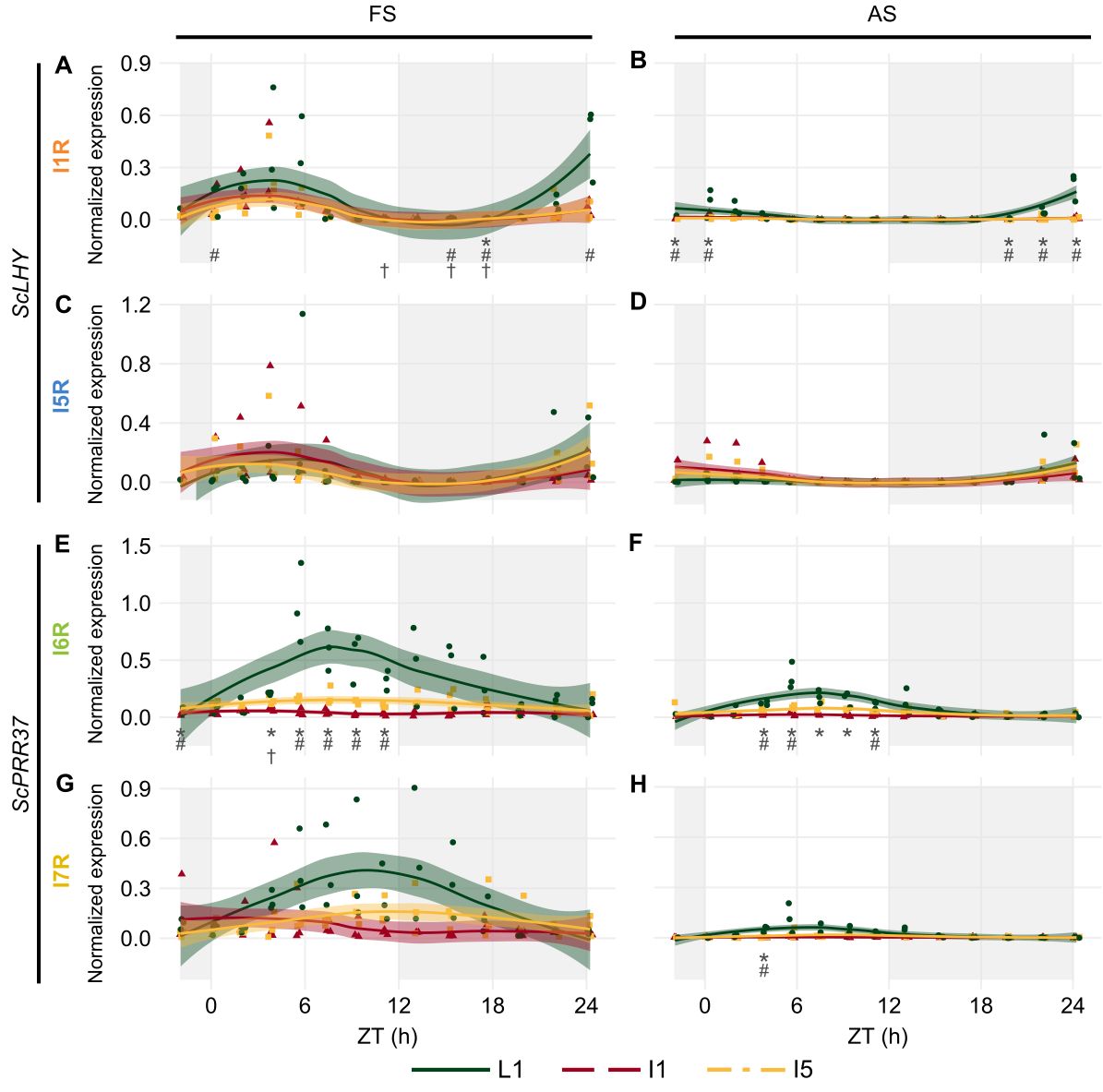

Supplement: Supplementary file 10 [file Image_8.jpeg]
